# Low 2D:4D Values Are Associated with Video Game Addiction

**DOI:** 10.1371/journal.pone.0079539

**Published:** 2013-11-13

**Authors:** Johannes Kornhuber, Eva-Maria Zenses, Bernd Lenz, Christina Stoessel, Polyxeni Bouna-Pyrrou, Florian Rehbein, Sören Kliem, Thomas Mößle

**Affiliations:** 1 Department of Psychiatry and Psychotherapy, Friedrich-Alexander-University of Erlangen-Nuremberg, Germany; 2 Criminological Research Institute of Lower Saxony, Hanover, Germany; Middlesex University London, United Kingdom

## Abstract

Androgen-dependent signaling regulates the growth of the fingers on the human hand during embryogenesis. A higher androgen load results in lower 2D:4D (second digit to fourth digit) ratio values. Prenatal androgen exposure also impacts brain development. 2D:4D values are usually lower in males and are viewed as a proxy of male brain organization. Here, we quantified video gaming behavior in young males. We found lower mean 2D:4D values in subjects who were classified according to the CSAS-II as having at-risk/addicted behavior (n = 27) compared with individuals with unproblematic video gaming behavior (n = 27). Thus, prenatal androgen exposure and a hyper-male brain organization, as represented by low 2D:4D values, are associated with problematic video gaming behavior. These results may be used to improve the diagnosis, prediction, and prevention of video game addiction.

## Introduction

A high prenatal androgen load, induced either by enhanced hormone levels or more sensitive androgen signal transduction pathways, results in a longer fourth digit (4D) relative to the second digit (2D) in the adult human hand [1]. Therefore, 2D:4D values are considered to be sexually dimorphic, with values usually lower in males compared with females [2]–[4]. Additionally, the prenatal androgen load has an organizing effect on brain structure and function [5]. As a result, 2D:4D values are associated with a wide range of male/female behavioral phenotypes. Low 2D:4D values are associated, for example, with autistic features [6], [7]; attention deficit hyperactivity disorder (ADHD) [8], [9]; athletic performance [10], [11]; spatial abilities [12]–[15]; abstract reasoning [16]; numeric abilities [17]–[19]; cooperativeness, pro-social behavior, and fairness [20], [21]; number of life-time sexual partners [22]; and reproductive success [23]. The evidence linking the prenatal androgen load with low 2D:4D values and behavioral traits has recently been reviewed [24], [25].

We have previously shown lower mean 2D:4D values in patients with alcohol dependence [26], a substance-related addictive disorder with a higher prevalence in males than females [27], [28]. In this study, we aimed to analyze whether low 2D:4D values are also associated with addictive video gaming behavior, which is a non-substance-related addiction behavior. Severe gaming behavior occurs much more frequently in males compared with females [29]–[32] and is associated with sensation seeking [33] and ADHD [34]. Pathological video gaming may be viewed as a hyper-male behavior. Therefore, we hypothesized that males with pathological video gaming behavior may have been prenatally exposed to a higher androgen load, as indicated by their lower 2D:4D values.

## Methods

This study is part of the Finger-Length in Psychiatry (FLIP) project of the Erlangen Department of Psychiatry and Psychotherapy as well as the longitudinal interview study module of the project entitled “Internet and Video Game Addiction – diagnostics, epidemiology, etiopathogenesis, treatment and prevention” of the Criminology Research Institute of Lower Saxony. The FLIP-Project was realized as an add-on at the second measurement occasion (t2) of the longitudinal interview study. This investigation has been conducted according to the principles expressed in the Declaration of Helsinki. The study was approved by the local ethics committee (Ethics Committee of the German Psychological Society [Deutsche Gesellschaft für Psychologie]). Written informed consent was obtained after providing a complete description of the study to all of the subjects.

Between February and December 2011, 70 subjects participated at the first measurement occasion (t1) of the longitudinal interview study (they were originally chosen from an overall 1,092 prospective participants which were recruited via schools, universities, internet forums, newspapers, and counseling centers). Prerequisites for study participation at t1: male, 18-21 years old, habitual video gamers with either more than 2.5 hours of gaming a day or a Video Game Addition Scale (CSAS-II) score > 41 [29], see below). From March 2012 to January 2013, 64 participants could be interviewed again at the t2 follow-up of the longitudinal interview study. At this measurement occasion a total of 54 subjects agreed to additionally participate in the FLIP project. These 54 subjects can be characterized as follows: 53 Caucasian, 1 Asian. Mean age at t1 was 18.9 years (*SD* = 1.1). 24 of the participants had a higher educational level (Abitur or higher), another 24 had secondary schooling (Realschule), 5 reported lower secondary schooling (Hauptschule) and one no graduation.

Video game addiction was assessed using the CSAS II [29] at t1. The CSAS II is based on the Internet Addiction Scale ISS-20 [35], [36], which has been extended and adapted to assess video game addiction. The CSAS-II consists of 14 items (4-point scale: 1 * =  incorrect* to 4 * =  absolutely true*) and covers the dimensions *preoccupation/salience* (4 items), *conflict* (4 items), *loss of control* (2 items), *withdrawal symptoms* (2 items), and *tolerance* (2 items). The items of the CSAS-II show high face validity, and the instrument demonstrates good convergent validity for subjective self-evaluation measures of video game addiction [29], [30]. Additionally, the CSAS-II-classification of video game addiction is not only associated with excessive gaming behavior but also identifies different measures of functional level and well-being [29], [30], [37]. The following diagnostic cut-offs are used: 14–34 =  unproblematic, 35–41 = at risk of becoming addicted, and 42–56 =  addicted.

According to CSAS-II classification, which is going beyond mere gaming times, 27 participants were classified as unproblematic video gamers, 17 as at risk of becoming addicted and 10 as addicted. Because of the small number of subjects investigated, the two groups “at risk of becoming addicted” and “addicted” were joined for analyses. Thus, two CSAS-II categories (unproblematic vs. at risk/addicted) with each 27 subjects were investigated in this study.

Psychological problems and symptoms of psychopathology were assessed at t1 using the Brief Symptom Inventory (BSI) [38]. The subscales interpersonal sensitivity (*T* = 52.26, *SD* = 11.81), depression (*T* = 53.98, *SD* = 11.64), anxiety (*T* = 54.30, *SD* = 10.23), and hostility (*T* = 52.20, *SD* = 11.56) were used as control variables in the multivariate analyses. In addition, ADHD symptomatology, which was also used as control variable, was assessed using the ADHD-Screening for adults (ADHS-E; *T* = 54.02, *SD* = 8.79) [39].

An Avision IS1000 flatbed scanner (Hsinchu, Taiwan) was used to scan the participants’ hands at t2. To increase accuracy, small marks were drawn on the basal creases of each of the participants’ index and ring fingers before scanning. Both hands were scanned at the same time, with palms down, in black-white mode. We used the GNU Image Manipulation Program (GIMP, version 2.8.4; www.gimp.org) to measure the lengths of the index (2D) and ring (4D) fingers from the hand scans. This technique provides good reliability [40]. The total length of the second and fourth digit of the left and right hands was quantified from the middle of the basal crease to the tip of the finger and was determined in units of pixels using the GIMP “measure” tool. The measurements were performed by three independent individuals who were blind to the hypothesis and blind to the diagnostic category. Mean values of the three measurements were calculated for the second and fourth digit.

Statistical analyses were computed using IBM SPSS 19 (Armonk, New York, USA) and the R software.

## Results

Differences in age between the unproblematic and at risk/addicted groups were analyzed by the Student’s t-test; differences in educational level by the Fisheŕs exact test for contingency tables larger than 2×2 [41], [42]. Both of the CSAS II groups (unproblematic vs. at risk/addicted) were well matched with respect to age (*t* = 1.544, *p* = 0.129) and educational level (*p* = 0.381; see Table 1).

**Table 1:** Mean 2D:4D and Dr–l values in individuals with unproblematic vs. at-risk/addicted video gaming behavior.

	unproblematic (n = 27)		at-risk/addicted (n = 27)	
	mean ± *SD*	min – max	mean ± *SD*	min – max
2D:4D mean	0.979±0.024	0.939 – 1.029	0.966±0.024	0.929 – 1.009
2D:4D right	0.977±0.025	0.928 – 1.030	0.967±0.018	0.942 – 1.011
2D:4D left	0.982±0.025	0.926 – 1.034	0.966±0.020	0.918 – 1.017
Dr–l	–0.0051±0.0157	–0.0354 – 0.0302	0.0012±0.0142	–0.0165 – 0.0358
CSAS	26.2±4.3	18 – 33	40.5±5.3	35 – 51
Age (years)	19.1±1.2	18 – 21	18.6±0.9	18 – 21
				
Level of Education				
	n		n	
Higher educational level (*Abitur* or higher)	14		10	
Secondary schooling (*Realschule*)	12		12	
Lower secondary schooling (*Hauptschule*)	1		4	
No graduation	0		1	

Dr–l =  right 2D:4D – left 2D:4D.

The reliability of the three measurements of the fingers was calculated for each finger separately for the right and left hand using the two-way random intra-class correlation coefficient (ICC) [43]. ICCs were also calculated for 2D:4D ratios and right 2D:4D–left 2D:4D (Dr–l) values. The reliability of the three raters was high for both the right hand (2D: ICC  = 0.995; 4D: ICC  = 0.995; 2D:4D: ICC  = 0.944), the left hand (2D: ICC  = 0.996; 4D: ICC  = 0.994; 2D:4D: ICC  = 0.937), and the arithmetic mean (2D:4D: ICC  = 0.961). The reliability of the Dr–l values was also high (ICC  = 0.764).

Deviation from normal distribution was tested by the Kolmogorov-Smirnov test. The 2D:4D (arithmetic mean: *Z* = 0.931, *p* = 0.351, left hand: *Z* = 0.550, *p* = 0.923, right hand: *Z* = 0.913, *p* = 0.375) and Dr–l (*Z* = 1.082, *p* = 0.193) values did not deviate from a normal distribution. The mean 2D:4D and Dr–l values are presented in Table 1.

Differences in 2D:4D and Dr–1 values depending on educational level were tested for the unproblematic and at risk/addicted group by the Kruskal Wallis test. Pearson correlation coefficients were calculated. The correlation between 2D:4D values for the right vs. left hand was 0.788 (*p* < 0.01). 2D:4D and Dr–l values did not differ significantly depending on educational level within the unproblematic (arithmetic mean: *χ^2^*(2, *N* = 54)  = 1.831, *p* = 0.400, left hand: *χ^2^*(2, *N* = 54)  = 2.247, *p* = 0.325, right hand: *χ^ 2^*(2, *N* = 54)  = 2.005, *p* = 0.367, Dr–1: *χ^2^*(2, *N* = 54)  = 0.637, *p* = 0.747) and at risk/addicted group (arithmetic mean: *χ^2^*(3, *N* = 54)  = 3.363, *p* = 0.339, left hand: *χ^2^*(3, *N* = 54)  = 2.139, *p* = 0.544, right hand: *χ^2^*(3, *N* = 54)  = 3.348, *p* = 0.341, Dr–1: *χ^2^*(3, *N* = 54) = 0.460, *p* = 0.928).

Associations between measures of 2D:4D (left hand, right hand, arithmetic mean, Dr–1) and video game addiction (unproblematic vs. at risk/addicted group) were tested by a non-parametric multivariate approach based on the principle of recursive partitioning, i.e. conditional inference trees (C-Tree; [44], [45]). Controlling for interpersonal sensitivity, depression, anxiety, hostility and ADHD, comparable to a stepwise regression non-significant predictors are excluded. Using the C-Tree algorithm the global hypothesis of independence between any of the input variables and the response variable is tested using a permutation test framework [46]. For metric variables the C-Tree algorithm implements a binary split in the selected input variable. To determine the “best” binary split, several split criteria are provided (e.g., “Gini importance”, “impurity of node” or “entropy”). However, most splitting criteria are not applicable to correlated response variables or response variables measured with different scale formats (e.g., metric and nominal). We therefore utilized the permutation test framework described by Hothorn et al. [47] (p. 6, equation 3). Since permutation tests derive the p-values from sample-specific permutation distributions of the test statistics, only p-values are reported. The R package “party” (a laboratory for recursive partitioning; [47], [48]) was used for this analysis.

**Figure 1:**
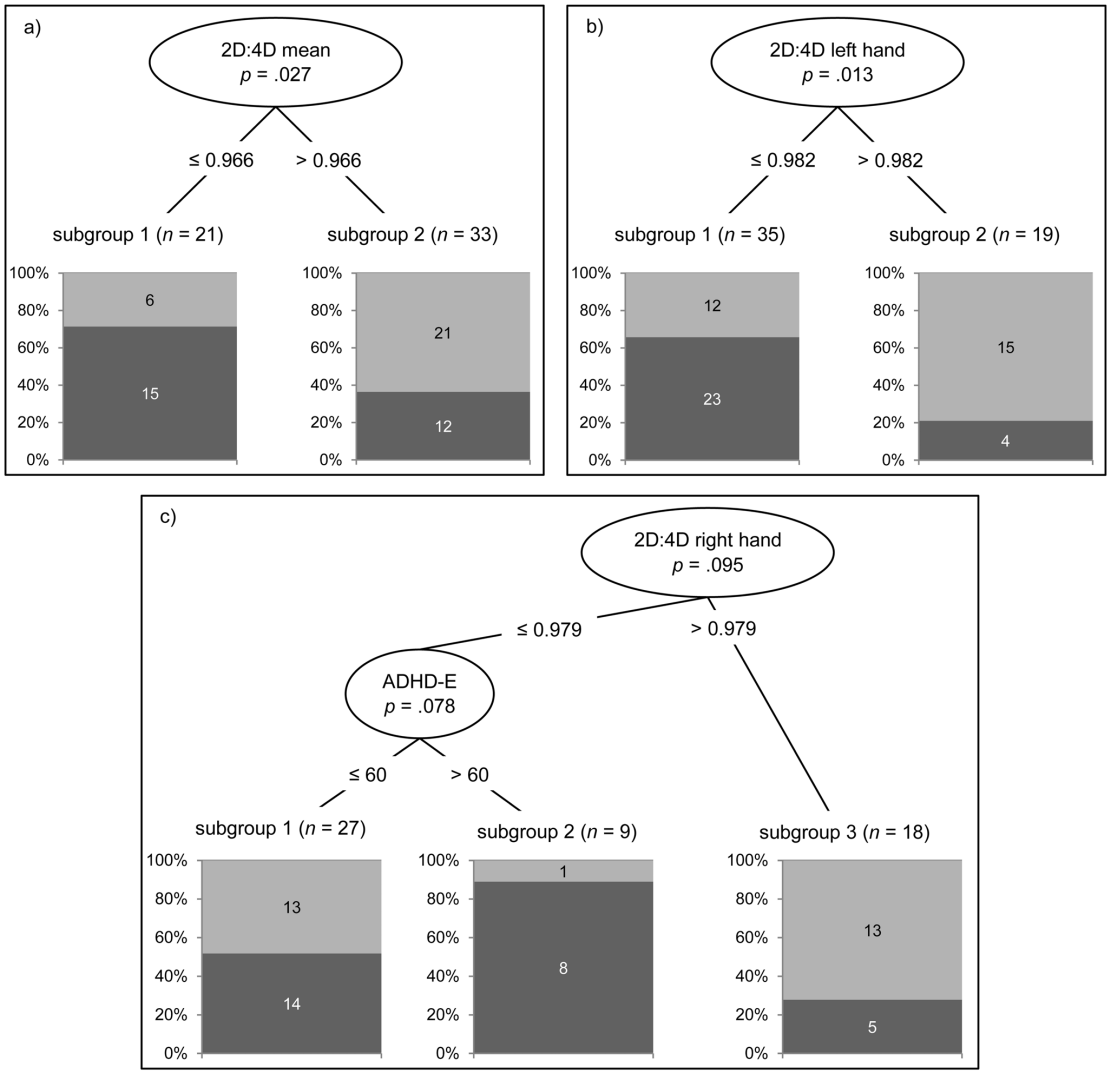
Conditional Inference tree plots. The risk of being video game addicted/at risk with respect to measures of 2D:4D. The percentage share of addicted/at risk participants in each subgroup is marked in black.

**Figure 2:**
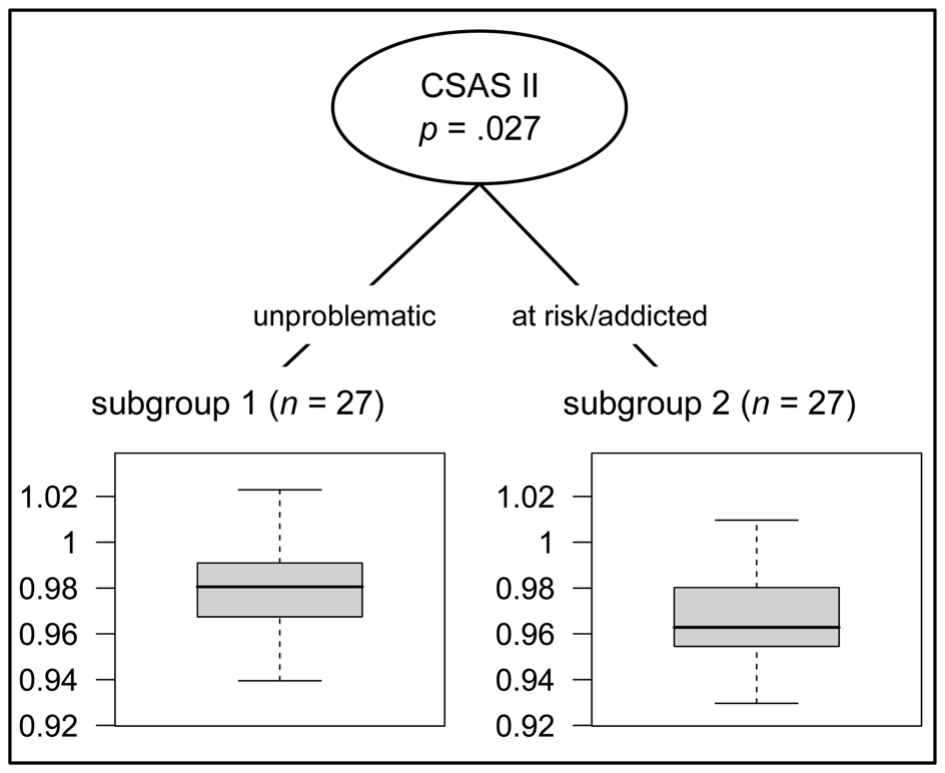
Conditional Inference tree plot. Box Plot of mean 2D:4D group differences between unproblematic and at risk/addicted gamer.

In the multivariate non-parametric analyses, measures of 2D:4D (arithmetic mean, left hand, right hand) were associated with video game addiction (unproblematic vs. at risk/addicted group) when controlling for interpersonal sensitivity, depression, anxiety, hostility and ADHD: 1. Study participants with a mean 2D:4D ratio lower than 0.966 showed a significantly higher risk of being video game addicted (*p* = 0.027, *d* = 0.71). 2. For the left hand, study participants with a 2D:4D ratio lower than 0.982 showed a significantly higher risk of being video game addicted (*p* = 0.013, *d* = 0.93). 3. For the right hand study participants with a 2D:4D ratio lower than 0.979 showed a significantly higher risk of being video game addicted on the level of *p* < 0.10 (*p* = 0.095, *d* = 0.66). Moreover, study participants who additionally scored higher than 60 (T-score) on the ADHS-E were particularly at risk (*p* = 0.078, *d* = 0.69). No significant association was found for Dr–1 (*p* = 0.127). Figures 1a to 1c illustrate the risk of video game addiction for the mean 2D:4D, as well as the left and right 2D:4D values in C-Tree. Independent of the reported 2D:4D cut off values mean group differences in measures of 2D:4D between unproblematic and at risk/addicted can be observed, which is exemplified for mean 2D:4D in figure 2 using the same analysis with reversed dependent and independent variables. Together, these results indicate that at-risk/addicted video gamers have smaller 2D:4D ratios.

To estimate the value of the 2D:4D ratio as a diagnostic test for the discrimination of video game-addicted/at risk individuals vs. controls with unproblematic gaming behavior, we used a ROC analysis to calculate AUC values, as well as sensitivity and specificity at the Youden point [49] (the point on the ROC curve where the sum of sensitivity and specificity is maximized). The ROC analysis shows that the diagnostic accuracy of the 2D:4D ratio of the left hand is highest (AUC 0.704, sensitivity 0.852, specificity 0.556), followed by that of the right hand (AUC 0.639, sensitivity 0.815, specificity 0.481). According to Hanley and McNeil [50] we checked for differences in paired AUCs with no significant result (*Z* = 1.147, *p* = 0.25).

## Discussion

This is the first investigation linking prenatal androgen exposure with addictive video gaming behavior. In this study, we found low mean 2D:4D values in subjects with at risk and addicted video gaming behavior. Effect sizes larger than d = 0.66 point to a moderate to strong effect [51]. No other considered predictors, except symptoms of ADHD for the right 2D:4D calculations were statistically significant in the multivariate nonparametric analyses. The observed association between at-risk/addicted video gaming and low 2D:4D values can be interpreted in several ways. (1) A small 2D:4D value directly induces addictive gaming behavior; however, there is no evidence in the literature to support this possibility. (2) Addictive gaming behavior directly induces low 2D:4D values. However, this possibility is unlikely because previous studies have proven that 2D:4D values remain constant throughout life after birth [52]. (3) A common mechanism is responsible for both low 2D:4D values and addictive gaming behavior. Based on the existing data, such a factor provides the most likely explanation. The results of the 2D:4D C-tree calculations with an additional explanatory power of symptoms of ADHD also support this explanation. Addictive gaming is more frequent in males [29]–[32] and is associated with ADHD [34] and sensation seeking [33]. All of these features have previously been linked to low 2D:4D values. One common reason for these associations appears to be a high androgen load during pregnancy.

Understanding the pathways leading from enhanced prenatal testosterone to game addiction will be crucial for defining potential policies targeting video game addiction. Prenatal testosterone may induce addictive behavior through several channels including the following: (1) Prenatal testosterone abundance modulates the mesolimbic reward system [53] thereby potentially affecting addictive gaming behavior in adults. (2) The specific rules of the cyber world as compared to the real world might compensate limitations in social interaction abilities caused by high prenatal testosterone load. Higher fetal testosterone levels have been shown to reduce empathy and the capacity to decode emotional facial expression, i.e. to understand what other people think and feel [54]. In line with that, lower 2D:4D values were related to reduced empathy in males [55]. Moreover, a smaller 2D:4D is linked to more indiscriminate social suspicion [56]. Thus, high prenatal testosterone might cause interpersonal problems and social isolation and, thereby, entail pathological video gaming behavior as a coping strategy. (3) It is likely that the abilities that facilitate or impede computer use modulate a person’s risk of developing video game addiction. Thus, our results concur with previous findings linking low 2D:4D with Java-related programming skills and high 2D:4D values with computer-related anxiety [57].

Previously, we found low mean 2D:4D values in individuals with alcohol addiction [26], a substance-related addiction disorder. It is noteworthy that low 2D:4D values also occur in individuals with a video gaming addiction, which is a non-substance related addictive disorder that is more prevalent in males than females. This result underscores the similarity between substance-related addiction and internet gaming addiction [58]. According to the DSM-5, internet gaming disorder is included in the appendix as a subject for further research. The literature suggests a biological basis of computer and internet gaming addiction [59]–[61]. The results presented here provide further evidence for a biological basis of internet gaming addiction and, thus, offer an argument for its classification as an addiction disorder.

Many phenomena have been linked to low 2D:4D values, most of which are compatible with the hyper-male brain hypothesis. Thus, low 2D:4D values may be regarded as a proxy of the endophenotype “hyper-male brain organization”. However, the precise effect of a high prenatal androgen load on the life of an individual and on that individual’s future adult behavior must also depend on additional variables and influences. The specific behavioral phenotype evolving as a result of the hyper-male brain organization most likely depends on a myriad of genetic and environmental factors that are experienced over an individual’s lifetime. Therefore, the presence of low 2D:4D values does not suggest a specific diagnosis or prognosis for any single individual. However, knowledge of 2D:4D values may aid in improving an individual’s diagnosis and prognosis associated with different problematic behaviors and disorders when used in combination with other markers.

These results may have important implications for the diagnosis, prevention, and consequences of addictive gaming. A low 2D:4D value alone is not diagnostic of addictive gaming, but this factor may facilitate the diagnosis when used in conjunction with other markers. A low 2D:4D value may help to identify individuals who are at risk for future development of addictive gaming and, thus, may facilitate prevention. Several attempts have been made to predict the development of internet gaming addiction in individuals [62]–[67]. A low 2D:4D value is a novel trait marker; combined with other markers, its use may improve the prediction of the future development or the current diagnosis of internet gaming addiction. Such improved prediction models may enable the development of effective preventive strategies.

We investigated individuals in a narrow age range; furthermore, the mean age did not differ between the two groups. In previous studies, age was, if at all, only marginally associated with 2D:4D values [68]. Therefore, age was not considered in the non-parametric analyses. Notably, education level did not differ between the two groups investigated in this study.

In additional analyses we also checked for a possible non-monotonic relationship between measures of 2D:4D and video game addiction using the CSAS-II sum score, as this has been reported for example for measures of 2D:4D and altruism [69]. The linear regression analyses revealed no significant linear, quadratic or combined trend – also with logarithmic transformation of the arithmetic mean (see [69]). Furthermore, these results were confirmed by non-parametric regression analyses [70], [71]. Together these analyses support the assumption to regard video game addiction as a categorical construct with qualitative distinct categories (unproblematic vs. problematic, i.e. at risk/addicted), such as previously reported for alcohol addiction [72].

The time spent with video gaming alone does not define addiction. For the diagnosis “video game addiction” further criteria have to be met: preoccupation, withdrawal, tolerance, loss of control, and continued use despite negative consequences. A strength of this study is the composition of the participants. All of the participants spent some time each day with video gaming, but only half of the participants had additional criteria defining them being at risk/addicted (as assessed by CSAS-II). Our results thus define 2D:4D as a risk factor specifically related to video game addiction, not just to video game playing per se.

Several study limitations should be noted. We used a mono-centric, cross-sectional, case-control design, which allows the detection of associations only, without causal relationships. Additionally, we investigated only males, and the sample group was relatively small. The strong effect size of 2D:4D on video gaming addiction probably enabled the detection of group differences despite the relatively low number of subjects. In our previous study, we also found a strong effect size relating 2D:4D to alcohol addiction [26]. Because of the well-known sex differences in addictive behavior [5], future studies should include females, should include other ethnicities and should also include a larger sample size.
